# Psychometric Characteristics of a Patient Reported Outcome Measure on Ego-Integrity and Despair among Cancer Patients

**DOI:** 10.1371/journal.pone.0156003

**Published:** 2016-05-19

**Authors:** Gitta Kleijn, Lenneke Post, Birgit I. Witte, Ernst T. Bohlmeijer, Gerben J. Westerhof, Pim Cuijpers, Irma M. Verdonck-de Leeuw

**Affiliations:** 1 Department of Clinical Psychology, Vrije Universiteit, Amsterdam, The Netherlands; 2 Department of Spiritual Care, VU University Medical Center, Amsterdam, The Netherlands; 3 Department of Epidemiology and Biostatistics, VU University Medical Center, Amsterdam, The Netherlands; 4 Department of Mental Health, University of Twente, Enschede, The Netherlands; 5 Department of Otolaryngology / Head and Neck Surgery, VU University Medical Center, Amsterdam, The Netherlands; University of St Andrews, UNITED KINGDOM

## Abstract

**Purpose:**

To evaluate psychometric characteristics of a questionnaire (the Northwestern Ego-integrity Scale (NEIS)) on ego-integrity (the experience of wholeness and meaning in life, even in spite of negative experiences) and despair (the experience of regret about the life one has led, and feelings of sadness, failure and hopelessness) among cancer patients.

**Methods:**

Cancer patients (n = 164) completed patient reported outcome measures on ego-integrity and despair (NEIS), psychological distress, anxiety and depression (Hospital Anxiety and Depression Scale (HADS)), and quality of life (EORTC QLQ-C30 (cancer survivors, n = 57) or EORTC QLQ-C15-PAL (advanced cancer patients, n = 107)). Confirmatory Factor Analysis was used to assess construct validity. Cronbach’s alpha was used to assess internal consistency. Convergent validity was tested based on a priori defined hypotheses: a higher level of ego-integrity was expected to be related to a higher level of quality of life, and lower levels of distress, depression and anxiety; a higher level of despair was expected to be related to a lower level of quality of life, and higher levels of distress, depression and anxiety.

**Results:**

The majority of all items (94.5%) of the NEIS were completed by patients and single item missing rate was below 2%. The two subscales, labeled as Ego-integrity (5 items) and Despair (4 items) had acceptable internal consistency (Cronbach’s alpha .72 and .61, respectively). The Ego-integrity subscale was not significantly associated with quality of life, distress, anxiety, or depression. The Despair subscale correlated significantly (p <.001) with quality of life (r = -.29), distress (r = .44), anxiety (r = .47) and depression (r = .32).

**Conclusion:**

The NEIS has good psychometric characteristics to assess ego-integrity and despair among cancer patients.

## Introduction

According to Erikson’s theory, ego-integrity and despair are key topics in the eighth developmental stage, when people enter the final stage of life and reflect on the meaning of life and how they lived it. A person experiences ego-integrity, if he or she accepts his or her life cycle as something that had to be, feels connected to others, and experiences a sense of wholeness, meaning and coherence as he or she faces (the approach of) death. Achieving ego-integrity is supposed to be associated with achieving wisdom [1–4] and less death anxiety [5]. In contrast, a person experiences despair, if he or she experiences regret about the life he or she has led, and has feelings of sadness, failure and hopelessness. It is suggested that despair is related to psychological distress, depressive symptoms, loneliness and isolation [3].

Not only older people in the final stage of life are confronted with death, ego-integrity and despair, but people with a life-threatening disease as cancer as well [3]. Much is known about the impact of cancer and its effect on quality of life [6–8]. We also know that prevalence of distress and depression among cancer patients is high [9]. However, information on ego-integrity and despair is scarce and a valid questionnaire to assess ego-integrity and despair among cancer patients is lacking. Such a questionnaire is important, because it is hypothesized that patients who do not achieve ego-integrity and have a high level of despair can experience more psychological problems and death anxiety, have fewer personal and interpersonal resources for facing cancer, and are more vulnerable to developing depressive symptoms [3,5].

The Northwestern Ego-integrity Scale (NEIS)—a questionnaire targeting ego-integrity and despair -was developed earlier based on research in the general population [10]. Recently, Westerhof et al. [11] investigated the NEIS among the general population aged 50–95 and reported that the NEIS has a two factor structure comprising Ego-integrity and Despair.

The aim of the present study was to investigate the psychometric characteristics of the NEIS among cancer patients. The research questions were: 1) Is the NEIS feasible among cancer patients?, 2) Is the same factor structure found compared to the general population [11]?, 3) Is the scale reliable in a cancer population, and 4) Has the scale validity in a cancer population?. Convergent validity of the questionnaire NEIS was tested based on a priori defined hypotheses: a higher level of ego-integrity was expected to be related to a higher level of quality of life, and lower levels of psychological distress, depression and anxiety; a higher level of despair was expected to be related to a lower level of quality of life, and higher levels of psychological distress, depression and anxiety [3]. The results of this study are expected to contribute to the development of a patient reported outcome measure to evaluate ego-integrity and despair among cancer patients.

## Methods

### Subjects and procedure

From 2009 to 2014, cancer patients were recruited for two intervention studies, on the effectiveness of life review therapy targeting advanced cancer patients (n = 107), and on the effectiveness of autobiographic writing targeting cancer survivors (n = 57). The studies were approved by the Medical Ethical Committee of the VU University Medical Center Amsterdam. In the present study, we used baseline data (before start of the intervention) of the patient reported outcomes measures: the Northwestern Ego-integrity Scale (NEIS), the Hospital Anxiety and Depression Scale (HADS) and the quality of life questionnaires EORTC QLQ-C30 (EORTC QLQ-C30 (cancer survivors) or EORTC QLQ-C15-PAL (advanced cancer patients)). In both studies patients under 18 years of age or with psychotic behavior, severe cognitive dysfunction or insufficient mastery of the Dutch language were excluded.

### Study measures

Patients completed a study specific questionnaire on sociodemographic characteristics (age, gender, marital status (married, widowed, divorced), children (number), and educational level (none, primary education, secondary general or vocational education, higher general or vocational education or academic education)), and clinical characteristics (treatment intent (curative or advanced cancer) and type of cancer (according to the ICD-10)).

The Northwestern Ego-integrity Scale (NEIS) [10, 12] is a 15-item questionnaire to assess despair and ego-integrity, with higher mean scores indicating more despair and ego-integrity [13]. The NEIS reflects Erikson’s conception of the eighth and final developmental phase in a person’s life, regarding ego-integrity and despair [1]. Respondents are asked to indicate their agreement to statements such as, ‘I have reached a point where I can accept the events in my life as having been necessary’ or ‘I wish I had more time to take a different path in life’ on a scale from 1 (strongly disagree) to 6 (strongly agree). A recent study among the general population aged 50–95, showed that the NEIS has 2 subscales, Ego-integrity (5 items) and Despair (4 items) [11].

The Hospital Anxiety and Depression Scale (HADS) is a 14-item questionnaire [14]. The HADS consists of two subscales, depression (HADS-D; 7 items) and anxiety (HADS-A; 7 items). The two subscales are combined in one overall score on psychological distress (HADS-T; 14 items). Scores on each item range from 0–3, with a score on the subscales ranging from 0–21 and a total score ranging from 0–42.

The European Organisation for Research and Treatment of Cancer Quality-of-Life Questionnaire C30 (EORTC QLQ-C30) is a 30-item questionnaire assessing quality of life among cancer patients [15]. It consists of five functional scales (physical, role, cognitive, emotional, and social), three symptom scales (fatigue, pain, and nausea and vomiting), and a global quality of life scale. The European Organisation for Research and Treatment of Cancer Quality-of-Life Questionnaire PAL15 (EORTC QLQ-C15-PAL) is a shortened version of the EORTC QLQ-C30. It is developed as a 15-item questionnaire assessing quality of life of cancer patients in palliative care [16]. In the present study, the single item for global quality of life (identical in the QLQ-C30 and QLQ-C15-PAL) was used (How would you rate your overall quality of life during the past week?), which is scored on a 7-point scale ranging from 1, ‘very poor’, to 7, ‘excellent’. According to the EORTC guidelines, this scale was transformed to a 100-points scale with a higher score indicating a better quality of life.

### Statistical analyses

Descriptive statistics were used to investigate the number of missing items. Confirmatory Factor Analysis was used to analyze whether the factor structure found in Westerhof et al. [11] could be replicated. Criteria for an acceptable fit were: 1) Root Mean Square Error of Approximation (RMSEA) <.06 and Comparative Fit Index (CFI) and Tucker-Lewis Index-Non-Normed Fit Index ≥.9. Measurement invariance of the factor structure was investigated between advanced cancer patients and patients treated with curative intent. Internal consistency of the subscales was assessed by calculating Cronbach’s alpha coefficient. Sum scores of the NEIS subscales were computed by summing all standardized item scores and rescaling the total score to a percentage score. Spearman’s rank correlation coefficients were computed to examine the relationship between the NEIS subscales and quality of life, psychological distress, anxiety and depression. The statistical comparisons were two-sided and a p-value <.05 was considered statistically significant. SPSS (version 20, IBM Crop., Armonk, NY USA), Mplus version 6.11 [17] and R version 3.1.1. (www.r-project.org) with the package lavaan [18] were used for the analyses.

## Results

### Subjects

A total of 164 cancer patients (57 patients treated with curative intent and 107 advanced cancer patients) participated. Mean age of the patients was 60.6 years (range 29–86; SD = 9.6), and 38.4% were male. Patients characteristics are shown in Table 1.

**Table 1:** Demographic characteristics of participants.

	Total group (n = 164)		Advanced cancer patients (n = 107)		Cancer survivors (n = 57)	
Scale	Distribution	%	Distribution	%	Distribution	%
**Gender**						
*Male*	63	38.4	57	55.3	6	10.5
*Female*	101	61.6	50	46.7	51	89.5
**Age**						
*Mean (SD)*	60.6 (9.7)		62.75 (9.3)		56.68 (9.2)	
*Range*	29–86		31–86		29–73	
**Marital status**						
*Married*	99	60.4	75	70.1	24	42.1
*Divorced*	29	17.7	13	12.2	16	28.1
*Widowed*	12	7.3	7	6.5	5	8.8
*Never married*	24	14.6	12	11.2	12	21.0
**Children**						
*Yes*	134	81.7	91	85.0	43	75.4
*No*	30	18.3	16	15.0	14	24.6
**Level of education**						
*Academic education*	27	16.5	15	14.0	12	21.1
*Higher general or vocational education*	54	32.9	27	25.2	27	47.4
*Secondary general or vocational education*	52	31.7	35	32.7	17	29.8
*Primary education*	28	17.1	28	26.2	0	0
*None*	2	1.2	2	1.9	0	0
*Unknown*	1	0.6	0	0	1	1.8
**Religion**						
*Yes*	76	46.3	35	32.7	41	71.9
*No*	87	53	72	67.3	15	26.3
*Unknown*	1	0.6	0	0	1	1.8
**Tumor type**						
*Lung cancer*	66	40.2	66	61.1	0	0
*Head and neck cancer*	2	1.2	2	1.9	0	0
*Hematological cancer*	33	20.1	23	21.5	10	17.5
*Breast cancer*	32	19.5	5	4.7	27	47.4
*Other*	21	12.8	11	10.3	10	17.5
*Unknown*	10	6.1	0	0	10	17.5

### Missing items

Overall, the majority of all items (94.5%) of the NEIS were completed by patients. Regarding the single items, missing item responses ranged from .6% to 1.2%.

### Confirmatory Factor Analysis

Westerhof et al. [11] suggested a 9-item factor structure with two subscales for the NEIS (Table 2). Assuming uncorrelated factors the fit was nearly acceptable (χ^2^(27) = 62.0, *p* = .0001; RMSEA = .089; CFI = .919; TLI = .892) and improved little when assuming correlated factors (χ^2^ (26) = 56.4, *p* = .0005; RMSEA = .084; CFI = .930; TLI = .902). Only configural invariance (χ^2^(52) = 74.1, *p* = .023; RMSEA = .073; CFI = .905) and no metric invariance (Δχ^2^(7) = 27.1, *p* = .0003; ΔCFI = .086) could be proven between advanced cancer patients and patients treated with curative intent.

**Table 2:** Confirmatory Factor Analysis of the Northwestern Ego-integrity Scale, correlated factors were assumed.

	Item	EIS	DS	*M*	*SD*
6.	I have reached a point where I can accept the events in my life as having been necessary.	.75		3.4	1.7
7.	As I grow older, I understand people more.	.30		4.7	1.2
10.	I see a meaningful thread running through the many events in my life.	.66		3.5	1.5
13.	Even my sufferings have had meaning.	.69		3.7	1.6
15.	As I get older, my life story makes more sense to me.	.61		4.0	1.3
2.	It pains me to think about dreams and goals I have had that I did not fulfill.		.47	3.6	1.7
5.	I wish I had loved more in my life.		.72	3.3	1.7
8.	I am bothered by mistakes I have made in the past.		.47	3.0	1.5
14.	I wish I had more time to take a different path in life.		.64	3.3	1.7

EIS = Ego-integrity subscale, DS = Despair subscale, *M* = Mean, *SD* = standard deviation.

### Internal consistency

Cronbach’s alpha coefficient .72 for the Ego-integrity subscale and .61 for the Despair subscale.

### Subscale scores

To compute the scores of both subscales, all individual item scores were first transformed to Z-scores. Z-scores of items 6, 7, 10, 13 and 15 were summed for the subscale ‘Ego-integrity’, Z-scores of items 2, 5, 8, and 14 were summed for the subscale ‘Despair’. The obtained subscale ‘Ego-integrity’ ranged from -10.2 to 7.3 and the ‘Despair’ subscale ranged from 5.6 to 6.6. Final scores were obtained by rescaling the summed Z-scores as follows: Total score = ((sum Z-score—minimum sum Z-score) divided by (maximum sum Z-score—minimum sum Z-score)) times 100. The mean total score for the subscale ‘Ego-integrity’ was 58.1 (*SD* = 19.6) and for the subscale ‘Despair’ 45.1 (*SD* = 21.7).

### Convergent validity

The Ego-integrity subscale did not correlate significantly with quality of life, psychological distress, anxiety or depression. The Despair subscale correlated significantly (*p* <.001) and negatively with global quality of life (*r* = -.29), and positively with psychological distress (HADS-T score; *r* = .44), anxiety (HADS-A; *r* = .47) and depression (HADS-D; *r* = .32) (see Table 3).

**Table 3:** Convergent validity.

	Ego-integrity subscale		Despair subscale	
	Spearman	P-value	Spearman	P-value
QoL	.11	.18	-.29	<.001
HADS T	-.084	.29	.44	<.001
HADS A	-.005	.95	.47	<.001
HADS D	-.14	.072	.32	<.001

## Discussion

In the present study, we investigated psychometric characteristics of the NEIS, a patient reported outcome measure on ego-integrity and despair among cancer patients. The feasibility was good: the majority of all patients (94.5%) completed all items, missing item rate was below 2%. The factor structure of the NEIS consisting of two subscales ‘Ego-integrity’ (5 items) and ‘Despair’ (4 items) was confirmed in the present study among patients treated with curative intent as well as advanced cancer patients. We found acceptable internal consistency for Ego-integrity (*α* = .72), which is in accordance with earlier research in the general population [11–13,19], and somewhat lower internal consistency for Despair (*α* = .61).

In the present study, the scores on the Despair subscale correlated significantly and negatively with global quality of life, and positively with psychological distress, anxiety and depression, which confirms our hypothesis and earlier research [3]. However, in contrast to our hypotheses, the scores on the Ego-integrity subscale did not correlate significantly with quality of life, psychological distress, anxiety or depression. Janis [10] stated that ‘ego-integrity is a complex psychological state that involves a belief that life has been meaningful, a sense of contentment or satisfaction, and a feeling that one’s desires have been adequately met’. It may be that the construct of ego-integrity is more related to existential issues and not to quality of life (which also entails physical aspects of life, especially among cancer patients) or psychological distress or psychiatric disorders like depression and anxiety. The findings of Westerhof et al. [11] seem to support this. Their results showed that ego-integrity was related to well-being. They also found that more depressive symptoms are significantly related to more despair, but not to ego-integrity. Future research is needed to explore these associations, for example via qualitative research methods.

There are some limitations to our study. Our sample comprised patients participating in randomized controlled trials and our sample size was relatively small which may have affected correlations among items. Future research to test the hypotheses in a more representative sample would therefore be interesting. Also, In the present study both cancer survivors and patients with advanced cancer participated. Although the factor structure was comparable with 2 scales (Ego-integrity and Despair), the factor loadings on these scales differed between the two groups. It may well be that these groups differ in how they experience ego-integrity and despair. Further research is necessary to examine this.

In this study we investigated the feasibility, internal consistency, and validity. Furthermore, we used summed Z-scores instead of standardized scoring coefficients, because in general summed Z-scores are more accessible in clinical practice. However, further research is needed to determine cut-off scores, cross-cultural validity, and responsiveness of the NEIS. Cut-off scores, and responsiveness are also important in clinical practice. Furthermore, investigating cross-cultural validity is necessary so that the NEIS can be used in other cultural groups outside of the Netherlands. In future research the NEIS can be used to investigate ego-integrity and despair among cancer patients. More insight is needed in the prevalence of ego-integrity and despair, and the course from diagnosis and treatment to long-term survivorship or end-of-life. Also knowledge on factors that may influence ego-integrity and despair among cancer patients can be enriched, such as sociodemographic (age, gender, socio-economic status), clinical (as cancer type and stage, treatment modality, treatment intent), and psychological factors (such as perceived threat of cancer, optimism, anxiety and depression).

In clinical practice, addressing ego-integrity and despair among cancer patients does not seem to be standard practice yet, but could be an important aspect of psychological functioning. Therefore, more attention to ego-integrity and despair is important to be able to support the patients better. It is also important to develop and investigate more interventions that aim to improve ego-integrity and prevent despair. The NEIS can be used as a tool to investigate the effectiveness of these interventions targeting cancer patients.
